# Two-year outcomes after arthroscopic surgery compared to physical therapy for femoracetabular impingement: A protocol for a randomized clinical trial

**DOI:** 10.1186/s12891-016-0914-1

**Published:** 2016-02-04

**Authors:** Nancy S. Mansell, Daniel I. Rhon, Bryant G. Marchant, John M. Slevin, John L. Meyer

**Affiliations:** Renew Physical Therapy, Seattle, WA 98118 USA; Center for the Intrepid, 3551 Roger Brooke Drive, San Antonio, TX 78234 USA; Madigan Army Medical Center, 9040 Jackson Ave, Tacoma, WA 98433 USA; University of Southern California, Los Angeles, CA 90089 USA

**Keywords:** Femoracetabular impingement, Arthroscopic surgery, Physical therapy, Conservative management

## Abstract

**Background:**

As the prevalence of hip pathology in the younger athletic population rises, the medical community continues to investigate effective intervention options. Femoracetabular impingement is the morphologically abnormal articulation of the femoral head against the acetabulum, and often implicated in pre-arthritic hip conditions of musculoskeletal nature. Arthroscopic surgical decompression and non-surgical rehabilitation programs focused on strengthening and stability are common interventions. However, they have never been directly compared in clinical trials.

The primary purpose of this study will be to assess the difference in outcomes between these 2 commonly utilized interventions for femoracetabular impingement.

**Methods:**

The study will be a single site, non-inferiority, randomized controlled trial comparing two different treatment approaches (surgical and nonsurgical) for FAI. The enrollment goal is for a total of 80 subjects with a diagnosis of Femoracetabular impingement that are surgical candidates and have failed 6 weeks of conservative treatment. This will be a convenience sample of consecutive patients that are Tricare beneficiaries and seeking care at Madigan Army Medical Center. Patients that meet the criteria will be screened, provide written consent before enrollment, and then randomized into one of two arms (Group I = hip arthroscopy, Group II = physical therapy). Group I will undergo hip arthroscopy with or without labral repair. Group II will follow an impairment based physical therapy program consisting of 2 sessions per week for 6 weeks. The primary outcome will be the Hip Outcome Score and secondary measures will include the International Hip Outcome Tool and the Global Rating of Change. Measures will be taken at baseline, 6 months, 1 and 2 years. Hip-related healthcare utilization between both groups will also be assessed at the end of 2 years.

**Discussion:**

The current evidence to support both surgical and conservative interventions for femoroacetabular impingement is based on low-level research. To date, none of these interventions have been directly compared in a randomized clinical trial. Clinical trials are needed to help establish the value of these interventions in the management of femoracetabular impingement and to help define appropriate clinical pathways.

**Trial registration:**

NCT01993615 30 October 2013.

**Electronic supplementary material:**

The online version of this article (doi:10.1186/s12891-016-0914-1) contains supplementary material, which is available to authorized users.

## Background

Femoracetabular impingent (FAI) consists of abnormal morphology of the acetabulum and/or proximal femur. This can result in cartilage injury as well as tears to the acetabular labrum [1–3]. The type of abnormal morphology is often separated into three categories, Cam (femoral neck), Pincer (acetabulum) and combined based on where the abnormality lies. This condition is encountered more commonly in younger active patients, and especially males. Some think it plays a role in the development of osteoarthritis, placing it within a pre-arthritic hip disease (PAHD) classification.

Because the diagnosis is strongly based on changes in bone morphology, radiographic imaging plays a significant role in the diagnosis, prognosis and surgical treatment of FAI. Common radiographic findings thought to aid in the diagnosis include measures of acetabular retroversion (crossover sign [1], ischial spine sign [2], and posterior wall sign [3]) and femoral neck angles and morphology (alpha angle [4], and pistol grip deformity [5]). These measures also assist with the planning for surgical interventions, and some authors have even linked radiographic findings with prognosis of FAI, such as the amount of radiographic joint space narrowing [6]. However, others studies point out the poor diagnostic accuracy of radiographic findings in the diagnosis of hip pathology, [7] and the potentially high prevalence of labral tears found in the asymptomatic population (as high as 73 %) [8, 9]. Others report very little diagnostic value in some of these same radiographic signs related to acetabular retroversion (crossover, ischial, and posterior wall) [10, 11].

This helps bring to light the diagnostic controversy related to FAI. If many of these radiographic anatomical variations help guide treatment decisions, but lack validity, then understanding optimal treatments for FAI becomes more challenging. The surgical approach focuses on addressing the anatomical impairments thought to cause the pathology. It has been referred to as a “hip preserving” intervention [12], that may potentially mitigate the onset of degenerative osteoarthritis. However, there is no evidence to suggest that progression of joint degeneration will not occur beyond what is expected in a morphologically normal control group [13, 14]. Only cohort-level studies have been performed to date, and these have reported favorable outcomes only in the short term [15–17]. Others caution about the significant variation in reported clinical and radiographic outcomes after surgery [15]. Zaltz and colleagues [18] warn that “indications and limits of arthroscopy must be carefully defined” and more research is needed to identify variables associated with failure rates and poor outcomes. Despite the challenges and controversy related to diagnosis, and lack of clinical trials to support surgical efficacy, surgery for the correction of FAI in the U.S. has increased significantly in the recent decade [9, 10]. This represents an eighteen-fold increase between 1999 and 2009 [11].

Conservative management is typically the precursor to most elective orthopaedic surgeries. Invasive surgical interventions are usually considered after conservative approaches have failed. However, there is also a large gap in the efficacy research of non-surgical interventions for FAI. Only low quality studies exist, consisting primarily of case studies and expert opinion [19–22]. Conservative management can include physical therapy, activity modification and avoidance of provocative hip positions [22]. There is no research beyond expert opinion to guide conservative physical therapy approaches to optimal management of FAI.

At present there are no randomized controlled trials that have compared surgery to a conservative intervention (e.g., physical therapy) for the treatment of FAI. Therefore, the primary purpose is to determine if there is a difference in self-reported functional outcomes between arthroscopic surgery and a supervised physical program for patients with FAI out at 2 years. The secondary aim is to evaluate the differences in hip-related healthcare utilization and associated costs between subjects in each group during the 2-year period following treatment.

## Methods/design

The study will be a single site, non-inferiority, randomized controlled trial comparing two different treatment approaches (surgical and nonsurgical) for FAI.

Potential subjects will be identified through the standard referral process to the orthopedic surgery and/or physical therapy clinic. Patients are referred through the computerized Composite Healthcare System (CHCS) by providers within the Madigan Army Healthcare System. An orthopedic surgeon at Madigan Army Medical Center (MAMC) will confirm that the patient is a candidate for hip arthroscopy prior to being considered for participation in the study (as outlined in the Inclusion/Exclusion Criteria). Research investigators and/or research assistants will ensure that potential subjects meet all inclusion and exclusion criteria prior to consent. All subjects will provide written informed consent before participating in the study. The protocol received ethics approval by the Madigan Army Medical Center Institutional Review Board. All interventions provided in this study are considered standard of care and could be given to a patient as part of their treatment plan even if they were not a part of this study. An ethics review will be conducted by Madigan Army Medical Center and monitored by the US Army Medical Department Clinical Investigation Regulatory Office to ensure compliance with federal regulations for protection of human medical research subjects. Participants can request to discontinue the study at any point.

Following consent, the investigators and/or research personnel will gather demographic information and objective measures for all subjects. A subject number will be assigned for each subject; all information collected will include the subject number only. All subjects will also complete a Hip Outcome Score (HOS), International Hip Outcome Tool Score, Global Rating of Change, Self Motivational Inventory, Pain Body Diagram, pain report via the Numeric Pain Rating Scale, and the Pain Catastrophizing Scale. The primary outcome measure will be the HOS at 2 years.

Subjects will then be randomized into one of two arms (Group I = Hip Arthroscopy, Group II = Physical Therapy). The method of group assignment will be determined via sequentially numbered opaque sealed envelopes. To minimize the risk of predicting the treatment assignment of the next eligible patient, randomization will be performed in permuted blocks of two or four with random variation of the blocking number. Date of enrollment will be used as the baseline date for both groups. Surgery will typically be scheduled within the following 3 months and physical therapy will typically be initiated within 1 to 2 weeks.

Group I will receive arthroscopy surgery. The hip arthroscopy will consist of acetabular rim trimming, labral repair or debridement and femoroplasty, all as indicated based on the surgeon’s clinical judgment with input from pre-operative imaging, exam findings and intra-operative findings. Typical surgery time is approximately 2 h in duration; surgery time may fluctuate up to 60 min depending on the complexity of the surgery. Hip arthroscopy is typically a same-day surgery; however subjects may require an overnight stay at the discretion of the orthopedic surgeon. This is standard for how a patient would receive this intervention outside of the study.

Following surgery, subjects will be evaluated by a physical therapist in the MAMC outpatient physical therapy clinic within 7 days to begin post-operative rehabilitation (Additional file 1). Typical post-operative rehabilitation is completed within 6 months following surgery; however additional care may be required at the discretion of the physical therapist. Subjects within this group will follow up with a research investigator and/or research assistant at MAMC to complete outcome measures following surgery at 6 month, 1 year and 2 year time periods.

Group II will receive a standardized physical therapy program that is supervised in the clinic for 6 weeks. Subjects will participate in two 45-min physical therapy visits a week for 6 weeks, for a total of 12 sessions. The physical therapy treatment plan will be based on individual impairments identified during the initial evaluation (Additional file 2). Typical treatment for FAI includes hip mobilizations (Additional file 3) and therapeutic exercise (Additional files 4 and 5), however other treatment interventions may be implemented at the discretion of the physical therapist.

After 12 sessions of physical therapy, patients will be released from treatment. Subjects can follow-up with their primary care provider for any additional specialty care referrals, to include orthopedics and physical therapy. Subjects will be analyzed in the groups they were originally randomized to.

Subjects will follow-up with a research investigator or research assistant that is unaware of the treament allocation, in order to complete outcomes measures at 6 month, 1 and 2 year time periods following the initial physical therapy evaluation.

**Fig. 1:**
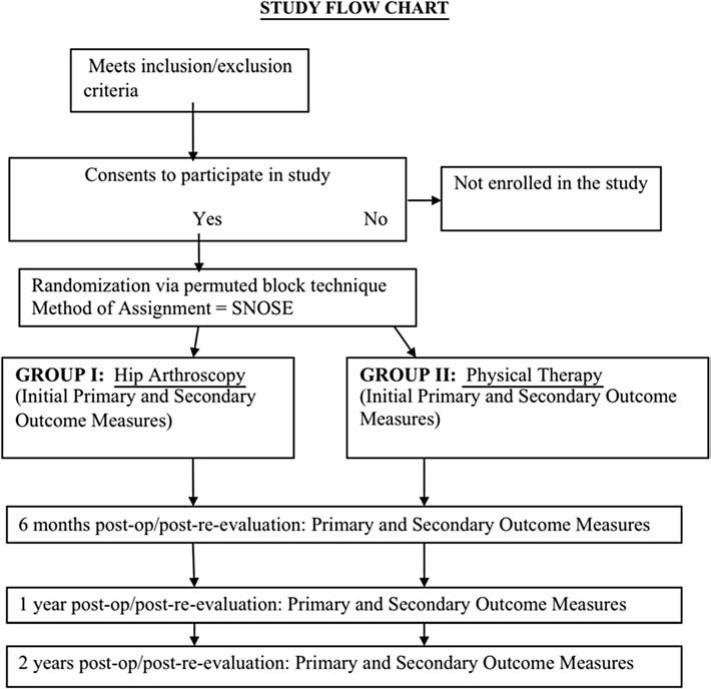
Proposed Recruitment Flow of Patients

This will be a single center randomized controlled trial with sequential enrollment of a convenience sample, with concealed allocation and blinded outcome assessors. The independent variable is treatment (physical therapy or arthroscopic surgery) and time with 4 levels out to 2 years. We will analyze any potential covariates that may affect prognosis, to include internal motivation and psychosocial beliefs using the Self Motivation Inventory scale and the Pain Catastrophizing Scale. This will be a trial assessing pragmatic delivery of two common interventions, and will report results following CONSORT guidelines for pragmatic trials [23]. The current SPIRIT guidelines for creating protocols for randomized clinical trials were followed in deriving the protocol [24]. Figure 1 demonstrates the flow of subjects through the trial.

### Participants

We plan to enroll 80 subjects into this study. Consecutive patients seen in the orthopaedic surgery clinic with hip pain that meet all inclusion/exclusion criteria will be offered the opportunity to enroll in the study. Therefore all subjects will be recruited from within the orthopaedic surgery service at Madigan Army Medical Center, after diagnosis and surgical candidacy for FAI has been established.

### Inclusion criteria (must meet all 3)

- Tricare beneficiaries between the ages of 18 and 60
- Must have a clinical diagnosis of FAI and/or labral pathology confirmed by a combination of all the following physical examination findings:

- ○Patient self-report of pain in the anterior hip or groin
- ○Pain reproduced with passive or active flexion
- ○Positive FADIR (Flexion Adduction Internal Rotation) test
- ○Subjective relief of pain after intra-articular injection

- Must be a surgical candidate for hip arthroscopy defined by (must have all 3):

- ○No less than 2 mm of joint space based on imaging (CT scan, radiographs and MR arthrogram)
- ○Positive crossover sign and/or alpha angle >50 deg based on imaging (CT scan, radiographs and MR arthrogram)
- ○Failed 6 weeks of conservative management

- ▪Conservative management includes NSAIDs, profile, patient education and exercise handouts

### Exclusion criteria

- Diagnosis of hip osteoarthritis more likely (joint space narrowing less than 2 mm.)
- Other concurrent systemic disease that may affect the condition (cancer, rheumatoid arthritis or systemic arthralgia/arthritis)
- Pending litigation/workmen’s compensation
- Will be moving or relocating within the following 6 months
- Clearing the lumbar spine reproduces the patient’s hip symptoms
- Pregnancy
- History of prior surgery on the same hip that will be analyzed in the study
- A formal course of physical therapy within the past 6 months
- Unable to give informed consent to participate in the study
- Unable to speak or read or write in English

### Randomization

Group assignment will be performed using sequentially numbered opaque sealed envelopes. Once the baseline examination is complete, an investigator blind to the randomization process will open the randomization envelope indicating subject’s treatment ground assignment. Subjects will be randomized into one of two arms (Group I = Hip Arthroscopy, Group II = Physical Therapy). A random-number generator will be used to establish randomization lists prior to the initiation of the study. To minimize the risk of predicting the treatment assignment of the next eligible patient, randomization will be performed in permuted blocks of two or four with random variation of the blocking number. Individual randomization assignments will be concealed according to the following procedure. The group assignment will be recorded on a 3.5X5 inch index card. This card will be folded in half such that the patient’s group assignment will be on the inside of the fold. The folded index card will then be placed inside the envelope and the envelope will be sealed. This will prevent the possibility of the investigator holding the envelope up to the light and visualizing the patient’s group assignment through a sealed yet potentially transparent envelope.

### Blinding

Based on the nature of this study, it is not possible to blind the patient or the clinician providing the intervention to the treatment received. However, the outcomes assessor will be blinded to treatment group at the follow-up time points.

### Interventions

Both treatment options are standard-of-care interventions. Their allocation and dosage are described in Table 1.

**Table 1:** Treatment Allocation and Dosage

Intervention	Subjects needed	Dosage	Follow up time (months)
Hip Arthroscopy	*N* = 40	One surgical session (to be followed by standard post-operative physical therapy by provider outside of this study)	6, 12, 24
Physical Therapy	*N* = 40	12 sessions with a physical therapist	6, 12, 24

### Hip arthroscopic surgery

The hip arthroscopy will consist of acetabular rim trimming, labral repair or debridement and femoroplasty, all as indicated based on the surgeon’s clinical judgment with input from pre-operative imaging, exam findings and intra-operative findings. Typical surgery time is approximately 2 h in duration; surgery time may fluctuate depending on the complexity of the surgery. Hip arthroscopy is typically a same-day surgery; however subjects may require an overnight stay at the discretion of the orthopedic surgeon. This is standard for how a patient would receive this intervention outside of the study.

### Physical therapy

At the first session, the examiner will perform a standardized clinical examination in order to develop a personalized impairment-based treatment plan (See Additional file 2). The six areas that will be tested include: anterior hip mobility (tested via the FABER position and Thomas test), hip flexion range of motion (ROM), prone and seated internal rotation ROM, lumbar mobility in the quadraped rock position, gluteus medius control in the lateral stepdown movement and proprioception and lower extremity neuromuscular control in the reverse lunge. Based on the specific impairments (reproduction of the primary hip pain complaint), patient’s supervised physical therapy program will incorporate joint mobilizations, mobilization with motion, therapeutic exercise, soft tissue mobility, stretching and motor control exercises to address these identified impairments (Additional files 3, 4, and 5) . The clinic program will be reinforced by a home exercise program to address the specific needs of the patient that will include 8–10 exercises by the conclusion of the 6 weeks.

### Outcome measures

The primary outcome will be the Hip Outcome Score. Secondary outcome measures will include the iHOT33, GROC, and NPRS. These will all be taken at baseline, 6, 12, and 24 months. The Self Motivation Inventory and Pain Catastrophizing Scale will also be taken at baseline and at 24 months. For our healthcare utilization analysis, we will collect healthcare utilization and associated costs that occurred for the duration of the study, and compare them between groups. We will be using the Military Health System Data Repository (MDR) for abstraction of data.

#### Hip outcome score (HOS)

The HOS is designed to assess higher level activities (i.e. those required in athletics) and has demonstrated validity in a study of patients at a mean of 3 years following hip arthroscopy [18]. Additionally, a 2011 study states that the HOS has the greatest amount of clinometric evidence (rigor of rating scales and indexes for the description of clinical phenomena). Only three published patient-reported outcome instruments utilized to assess FAI and labral pathology use clinometric evidence and the HOS was identified as the most proven instrument [25].

#### International hip outcome score (iHOT33)

The iHOT33 is a quality-of-life patient-reported outcome measure that uses a visual analog scale response format for young, active patients with hip pathology. This 33-item questionnaire includes four sections: symptoms and functional limitations, sports and recreational activities, job related concerns and social, emotional and lifestyle concerns. This outcome measure has shown to be reliable; shows face, content and contrast validity and is highly responsive to clinical change [26].

#### Global rating of change (GROC)

The GROC will be administered to patients at the initial data collection, 6 months, 1 and 2 years. The GROC questionnaire is a common, feasible, and useful method for assessing short-term outcomes and overall changes in quality of life [27, 28]. The GROC has a 15-point scale with a change of positive three points or higher demonstrating clinically significant improvement in a patient’s perception of quality of life [27].

#### Self motivation inventory

The Self-Motivation Inventory is an outcome measure that was developed to determine level of motivation. The Self-Motivation Inventory is a 40-item tool that has been found to measure an individual’s tendency to persevere independent of situational reinforcement. It has also been used to predict successful weight loss and may correlate with number of sessions attended in weight loss program [29]. This tool underwent refinement in 1980 with the original 60-item tool being tested in undergraduate male and females. Items correlating less than 0.30 were deleted. The final 40-item scale yielded an exceptionally high internal reliability (α = 0.91) suggesting that a unitary common concept is evident for the obtained factor structure. The tool is widely used and has been tested in weight loss and therapeutic exercise studies [29].

#### Numeric pain rating scale (NPRS)

A 0–10 numeric pain rating scale (‘0’ indicating no pain, and ‘10’ worst imaginable pain) will be used to assess hip pain intensity. Numeric pain scales are known to have excellent test-retest reliability [30].

#### Pain catastrophizing scale (PCS)

The PCS is a 13-item patient-report scale developed to measure the extent to which people catastrophize in response to pain. Each item is scored from 0 (‘not at all’) to 4 (‘all the time’). The PCS is reported as a total score, with higher scores indicating greater catastrophizing, and is composed of three sub-scales: Rumination (four items; e.g. ‘When I am in pain, I keep thinking about how badly I want the pain to stop’), Magnification (three items; e.g. ‘When I am in pain, I become afraid that the pain will get worse’), and Helplessness (six items; e.g. ‘When I am in pain, I feel I can’t go on’) The PCS has been shown to have high levels of internal consistency and construct validity [31, 32].

### Justification of sample size

Our power analysis was based on the effect size reported by Martin and colleagues [33] for the Hip Outcome Score. They reported MCID values of 9 and 6 points for the Activities of Daily Living (ADL) and sports subscales respectively [33]. In order to detect a significant change between groups that is clinically meaningful, we will need to enroll 20 subjects per group based on the effect size of the ADL subgroup in the Martin study. This derived minimum sample size will provide us statistical power of 80 % with an alpha of 0.05. This is the more conservative analysis. However, the follow-up time period in the referenced study was only out to 6 months whereas this study will be out to 2 years. Accounting for 10 % loss to follow-up per year, which often happens in active duty military populations that deploy or get reassigned to multiple posts, we will plan on recruiting 80 subjects.

### Data analysis

The primary analysis of effectiveness will be performed using a linear mixed-effects model that is flexible in accommodating data that is assumed to be missing at random. There will be 4 time points for measurement of each outcome (baseline, 6, 12, and 24 months). Subjects will be analyzed in the groups that they were originally allocated to. Sensitivity analyses will be performed to assess the impact of treatment cross over in the results. Descriptive statistics will be computed to characterize and compare the 2 treatment groups for assessment of baseline heterogeneity. Distributions of measured variables will be examined visually with frequency histograms and formally assessed with Kolmogorov-Smirnov and Shapiro-Wilk statistics to test the normality assumption. Levene’s test will be used to assess for violations of the homogeneity of variance assumption. Baseline comparability of groups will be tested with chi-square statistics for categorical variables and with unpaired t-tests if parametric assumptions are satisfied; Mann–Whitney U tests otherwise. All statistical tests will be performed at an alpha level of 0.05.

If one treatment is shown to be superior to the other, supplemental analyses will be performed by dichotomizing groups based on minimal clinically important differences (MCIDs) of 9 for HOS and +3 points for GROC scores. This will allow computation of absolute risk reduction, relative risk reduction, and number needed to treat (with associated 95 % confidence intervals) using failure to obtain clinically meaningful benefit as the event of interest.

Healthcare Utilization will be extracted from the Department of Defense Military Health System Data Repository (MDR) database which is the most commonly used system operated by TRICARE for research, containing direct and purchase care data. The amount and type of healthcare associated with an FAI-related diagnosis, along with related cost, will be abstracted for every patient in the study. This will include diagnostic imaging tests, medications, and specialty referrals. The amount of utilization for each category will be compared between groups.

### Trial organization and monitoring

The investigative team includes the authors listed in this protocol. The principal investigator will manage data flow and perform audits of the procedures, enrollment and treatment throughout the entire process of this study. The associate investigators will monitor the data-collection process and data integrity with periodic evaluation performed continually during the course of the data-collection phase.

## Discussion

The current evidence to support both surgical and conservative interventions for femoroacetabular impingement is based on low-level research. None of these interventions have been compared in a randomized clinical trial. This is likely due in part to the challenging methodology associated with conducting trials of this nature. These treatments are often considered sequential rather than parallel, meaning that often surgery is not considered an option until conservative treatment fails to achieve the desired effect.

Comparative studies of this nature have been conducted successfully in other body regions. Kirkley et. al. [34] compared arthroscopic surgery and physical therapy to only physical therapy in patients with moderate-to-severe knee osteoarthritis. Six of the patients assigned to surgery did not undergo surgery, and none of the patients randomized to physical therapy crossed over to have surgery. In contrast, a study by Katz and colleagues [35] had much higher cross over rates. They randomized 351 patients with knee osteoarthritis and a meniscus tear to receive either physical therapy or arthroscopic surgery. At 6 months, 51 (30 %) of the patients randomized to physical therapy had undergone surgery, while only 9 (6 %) that had been assigned to surgery had not had their surgery. Weinstein and colleagues [36] conducted two-tiered study consisting of a prospective cohort tier and a randomized controlled tier for patients with degenerative spondylolisthesis and lumbar spinal stenosis. The randomization tier placed patients into either a standard decompressive laminectomy or usual nonsurgical care. Patients were offered their choice of either the cohort (for those that definitely wanted surgery) or the randomization between surgery and physical therapy. There was a cross over rate of approximately 40 % for each group in the randomization tier.

Crossing over to receive a different treatment than was originally assigned is a common concern, especially in pragmatic trials. Although this may not reflect a sterile outcome from a specific treatment, the importance of analyzing subjects within the original groups they were randomized to, regardless of cross over, is an important step to minimize bias, and necessary if trials results are to be reported in adherence to the CONSORT statement for transparent reporting of clinical trials [37].

The challenge of recruitment is not limited to patient preference alone. Clinical equipoise describes the ethical challenges that healthcare providers face when assigning patients to various treatment arms of a clinical trial [38]. In this case, if a surgeon believes that the surgical arm is truly superior, it may pose an ethical dilemma when offering treatment choices to a patient. A study assessing the clinical equipoise of surgeons faced with clinical scenarios that involved randomizing patients to receive operative or non-operative treatment found that 77 % of the polled surgeons that routinely performed surgery for FAI were willing to recruit patients into a clinical trial that included a non-operative arm [39]. Seventy-five percent of the respondents felt that non-operative treatment ≥ 12 months would still be appropriate [39].

Due to the established prevalence of hip pathology, and the rising rates of surgery for FAI, there is a need to determine the impact of surgical and non-surgical treatments on long-term clinical results. A comparison of treatment options for FAI will help guide healthcare providers in decisions related to optimal effectiveness and timing of treatment.

## Additional files

Additional file 1:
**Post-Operative Rehabilitation Protocol.** (PDF 2770 kb) Additional file 2:
**Clinical Reasoning.** (PDF 666 kb) Additional file 3:
**Physical Therapy Program—Manual Therapy Approach.** (PDF 1678 kb) Additional file 4:
**Physical Therapy Program—Motor Control Exercises.** (PDF 3702 kb) Additional file 5:
**Physical Therapy Program—Mobility and Stretching Exercises.** (PDF 609 kb)

## Abbreviations

ADL: Activities of Daily Living
CHCS: Composite Healthcare System
CONSORT: Consolidated standards of reporting trials
DHA: Defense Health Agency
FABER: Flexion, abduction, and external rotation
FADIR: Flexion Adduction Internal Rotation
FAI: Femoracetabular impingement
GROC: Global Rating of Change
HOS: Hip Outcome Score
IHOT-33: International Hip Outcome Tool
MAMC: Madigan Army Medical Center
MCID: Minimal clinically important differences
MDR: Military Health System Data Repository
NSAIDs: Non-steroidal anti-inflammatory drugs
NPRS: Numeric Pain Rating Scale
PAHD: Pre-arthritic hip disease
PCS: Pain Catastrophizing Scale
ROM: Range of motion
SNOSE: Sequentially numbered, opaque sealed envelopes
SPIRIT: Standard protocol items: recommendations for interventional trials
