# Supplementary material for: Two-year outcomes after arthroscopic surgery compared to physical therapy for femoracetabular impingement: A protocol for a randomized clinical trial
Source: BMC Musculoskelet Disord. 2016 Feb 4;17:60. doi: 10.1186/s12891-016-0914-1 (PMC4743428; doi:10.1186/s12891-016-0914-1)
Supplement: Additional file 1: — Post-Operative Rehabilitation Protocol. (PDF 2770 kb) [file 12891_2016_914_MOESM1_ESM.pdf]

Hip Arthroscopy for FAI (Femoroplasty and Acetabuloplasty) Rehabilitation

Madigan Army Medical Center - Physical Therapy

	0 to 7 days Post-op	8 days to 21 days Post-op	22 days to 48 days Post-op	49 days Post-op
**Document**	NV status, pain, level, swelling, ecchymosis, gait status	NV status, pain level, swelling, ecchymosis, gait status, ROM, strength	Pain level, swelling, gait, ROM & strength, current exercise program	Functional level, ROM & strength, current exercise program
**Follow up**	Ortho 10-14 days post op for wound check and suture removal	Ortho 6 weeks post-op<br>Every 3-4 weeks with PT, attend PT 2-3x/wk	Ortho 12 weeks<br>Every 3-4 wks with PT, attend PT 2-3x/wk	Every 2-3 weeks with PT. Perform exercises 3-5x/wk. F/u w/ ortho PRN.
**Crutches**	WBAT x 3 weeks (MUST use crutches for stability)	WBAT x 3 weeks (MUST use crutches for stability)	FWB	FWB
**Precautions**	-NOTE: Do not push through hip flexor pain	-NO BALLISTIC/FORCED STRETCHING	NO BALLISTIC/FORCED STRETCHING, NO TREADMILL, NO CONTACT ACTIVITIES	None
**Wound**	-Check for Erythema/ Abnormal Temperature/ Excessive Effusion/ Drainage.	-Begin scar massage when incision site re-epithelializes	-Continue scar massage	n/a
**Exercises**	- Stationary bike w/ minimal resistance<br>- Seated piriformis stretch<br>- Glut/quad/HS/TRA isometrics<br>-<u>PROM Series</u>:<br>**(Continue Until DAY 22 Post-op)**<br>*SUPINE*:<br>1. Circumduction (CW/CCW)<br>2. Non-affected SLR (affected leg straight)<br>3. Affected SLR (Non-affected leg straight)<br>4. Circumduction knee bent (CW/CCW)<br>5. Hip flex (opposite leg bent)<br>6. FABER (let fall out, assist up)<br>7. Long axis abduction<br>8. Passive supine roll (long leg IR/ER)<br>*PRONE*:<br>1. IR<br>2. ER<br>3. Knee flex	- Double leg hip rotations<br>- Quadruped rocking<br>- Standing hip IR on stool<br>- Heel slides<br>- Hip abd/add isometrics<br>- Uninvolved knee to chest<br>- IR/ER (gentle to moderate resistance)<br><br>***<u>**Begin Standing AROM when patient can demonstrate symmetrical WB w/out assistive device**</u>:<br>- Double leg Romanian Dead Lift<br>- Double leg pelvic circles<br>- Lateral weight shift with overhead reach<br><br><u>**Begin post-op Day 15**</u>:<br>- Clam Shell<br>- Hip 3-way: abd/add/quadruped ext<br>- Bridge w/ tubing	**Continue previous exercises and ADD**:<br>- Kneeling hip flexor stretch<br>- Leg press (low weight)<br><br><u>**Begin post-op Day 29**</u>:<br>- Standing Figure 4 stretch<br>- Prone FABER position self-mobs<br>- Dyna-disc Single leg<br>- Side plank<br>- Standing hip IR (stool)<br><br><u>**Begin Post-op Day 36**</u>:<br>- Manual long axis distraction<br>- Bridge: single leg<br><br><u>**Begin Post-op Day 42**</u>:<br>- Elliptical<br>- Single leg trunk rotation with band<br>- Side stepping<br>- Slide board, side to side with push-off at ends<br>-Lateral step-down with heel hover	- Lunges<br>- Hurdles (slow speed)<br>- Carioca (slow speed with external ROT at 90 degrees hip flexion)<br>- Agility Ladder:<br>Forward double leg hop (land in mini-squat), Lateral hops (both directions)<br>Lateral shuffle (both directions):<br>-Plank to pushup start position<br>- Side stepping w/band<br>- Lateral step-downs w/ heel hover<br>- Isolateral Romanian Deadlift: partial range holding dowel along spine<br><br><u>**Begin Post-op Day 55**</u>:<br>-Multi-directional lunges<br>-Hurdles (medium to fast speed)<br>-Carioca (medium to fast speed with external ROT at 90 degrees hip flexion)<br>-Agility ladder: Single leg hops, Double leg hop 2 squares forward and 1 square backward<br>-Isolateral Romanian Deadlift (start and end position with contralateral LE in 90 deg hip flexion)<br>-Return to run progress as early as <u>**Post-op Day 63**</u>
**Modality**	-Game ready/ice and elevation	-Game ready/ice and elevation	-PRN for chronic swelling/scar formation	-Ice PRN
**Goals**	*1) Protect healing tissue*<br>*2) Restore ROM*<br>*3)Diminish pain/inflammation*<br>*4) Prevent muscular inhibition*<br>%	*1) Protect repaired tissue*<br>*2) Restore ROM/ normal gait pattern*<br>*4)Progressive increase in strength*	*1) Restore muscle endurance and strength*<br>*2) Restore cardiovascular fitness*<br>*3)Restore balance/proprioception*	Return to full duty when:<br>*1) Full ROM*<br>*2) Hip strength > 85% uninvolved side*<br>*3) Perform sport/job specific drills at full speed without pain*
