# Supplementary material for: Two-year outcomes after arthroscopic surgery compared to physical therapy for femoracetabular impingement: A protocol for a randomized clinical trial
Source: BMC Musculoskelet Disord. 2016 Feb 4;17:60. doi: 10.1186/s12891-016-0914-1 (PMC4743428; doi:10.1186/s12891-016-0914-1)
Supplement: Additional file 2: — Clinical Reasoning. (PDF 666 kb) [file 12891_2016_914_MOESM2_ESM.pdf]

Supplemental File A: Clinical Reasoning Framework

**Positive with Test or Movement**<br>***(Positive = Pain reproduced or asymmetry with opposite side)***	**Paired Treatment Approach for Manual Techniques**
FABER	• Prone FABER MWM<br>• Tri-planar kneeling self-mobs<br>• Standing Figure-4 Stretch<br>• Prone FABER self-mobs
Internal Rotation	• Internal Rotation MWM<br>• Half kneel CKC IR self-mobs with distraction
Quadruped Rock	• Quadruped MWM<br>• Quadruped Self-Mobs with distraction

MWM = Movement with Mobilization
FABER = Flexion Abduction External Rotation
