# Supplementary material for: Two-year outcomes after arthroscopic surgery compared to physical therapy for femoracetabular impingement: A protocol for a randomized clinical trial
Source: BMC Musculoskelet Disord. 2016 Feb 4;17:60. doi: 10.1186/s12891-016-0914-1 (PMC4743428; doi:10.1186/s12891-016-0914-1)
Supplement: Additional file 3: — Physical Therapy Program—Manual Therapy Approach. (PDF 1678 kb) [file 12891_2016_914_MOESM3_ESM.pdf]

SUPPLEMENTAL FILE D: Manual Therapy Mobilization Techniques

Note: All subjects have provided their consent to appear in the figures.

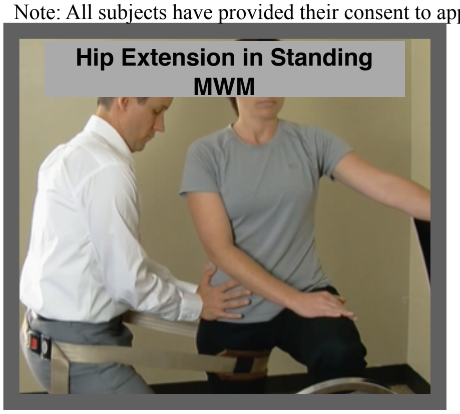

Clinician Position:
Standing next to the patient, on the side of the hip to be treated. Hands over ilium
Technique Description:
Apply a lateral glide as the patient leans forward into extension.

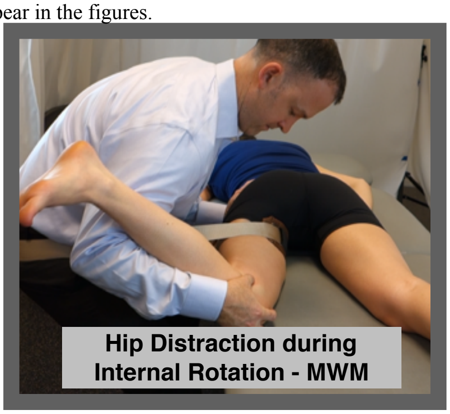

Clinician Position:
Standing next to patient, one hand on lateral hip and the other on distal thigh
Technique Description:
Apply a lateral glide as the patient's hip moves into internal rotation (passive and active)

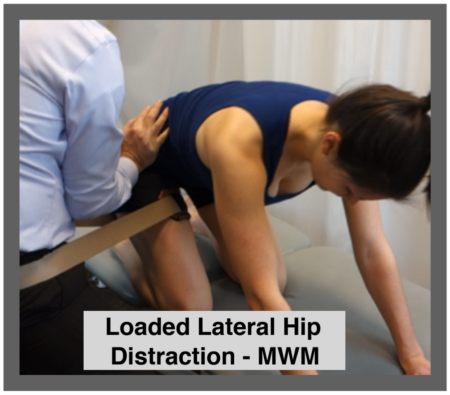

Clinician Position:
Standing at side of involved hip
Technique Description:
Have patient rock forward and back (into loaded hip flexion) while a lateral glide force is imparted

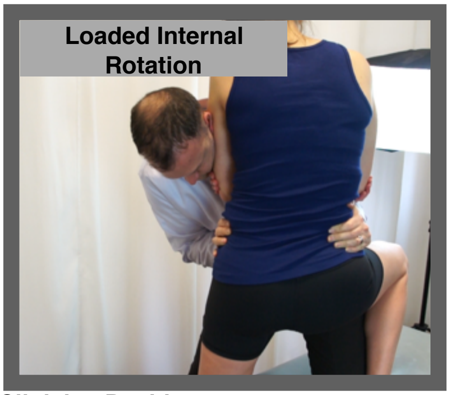

Clinician Position:
Stand facing the patient, Place a hand over each iliac crest of the patient. Use your thigh to block and stabilize the leg.
Technique Description:
Patient's knee on the side to be mobilized down and opposite hip up to put their foot flat on the table. Gently rotate the pelvis in towards you to provide a loaded internal rotation force to the left hip

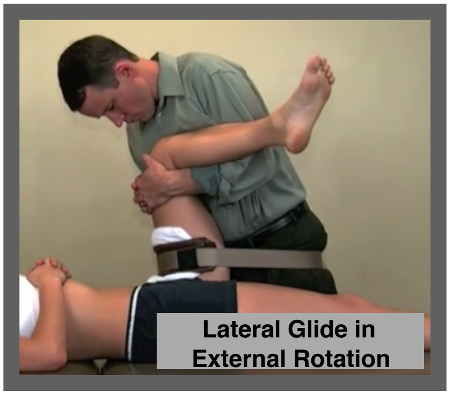

Clinician Position:
Standing facing patient's involved hip
Technique Description:
Maintaining the thigh perpendicular to the table, introduce hip external rotation until pain and/or resistance is perceived. Apply a distraction force by moving your hips/buttocks posteriorly. After mobilizing for 10-20 seconds, move the hip into further external rotation to engage the new restrictive barrier and repeat process.

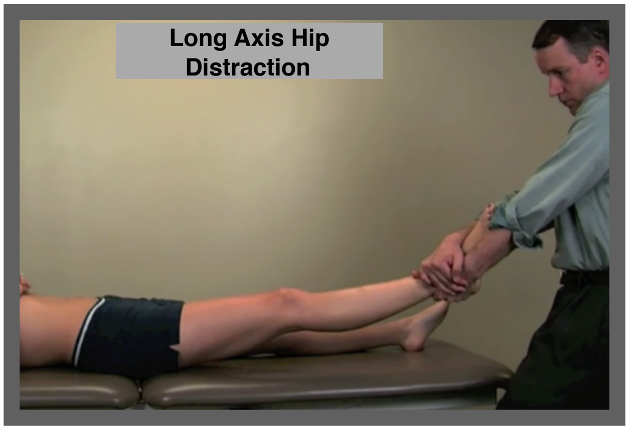

Clinician Position:
Standing at foot of patient, both hands around the ankle with elbows locked.
Technique Description:
Grasp the patient's ankle with both hands just above the malleoli. Position the hip in slight flexion and abduction
Apply a distraction force to the hip by shifting your weight to your back foot and pulling with both arm. The technique may be performed as a graded mobilization into resistance or as a high-velocity thrust at end range. Progress the technique by positioning the hip in further abduction and internal rotation prior to performing the mobilization or thrust manipulation
