# Supplementary material for: Two-year outcomes after arthroscopic surgery compared to physical therapy for femoracetabular impingement: A protocol for a randomized clinical trial
Source: BMC Musculoskelet Disord. 2016 Feb 4;17:60. doi: 10.1186/s12891-016-0914-1 (PMC4743428; doi:10.1186/s12891-016-0914-1)
Supplement: Additional file 4: — Physical Therapy Program—Motor Control Exercises. (PDF 3702 kb) [file 12891_2016_914_MOESM4_ESM.pdf]

SUPPLEMENTAL FILE B – Motor Control Exercises

Note: All subjects have provided their consent to appear in the figures.

<table>
<tr><th colspan="2">Exercise Name: Reverse Lunge with Front Ball Tap (gluteus focused)</th></tr>
<tr><td>Start Position</td><td>End Position</td></tr>
<tr><td>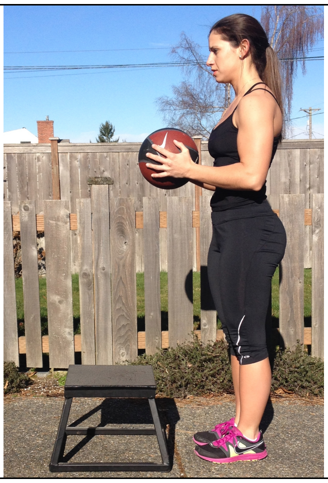</td><td>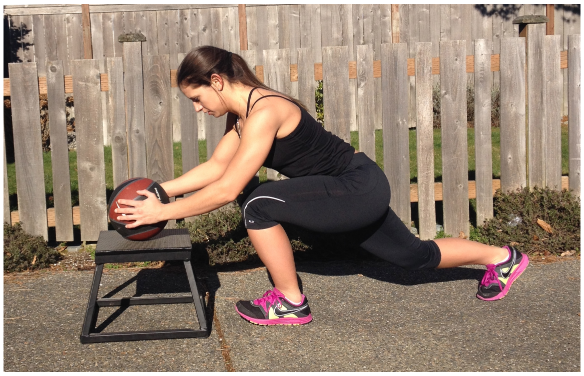</td></tr>
<tr><td colspan="2">1. Stand in a front of a medium sized box holding a medicine ball.
2. Take a step back while simultaneously placing the medicine ball on the box, bending the back knee.
3. Lift the toes of your front foot so that all of the weight is through your heel.
4. Lower the back knee and then return to starting position.
Dosing Guidance: 3 SETS of 10 repetitions performed 3-4 TIMES/WEEK</td></tr>
</table>

<table>
<tr><th colspan="2">Exercise Name: Isolateral Romanian Deadlift with Dowel</th></tr>
<tr><td>Start Position</td><td>End Position</td></tr>
<tr><td>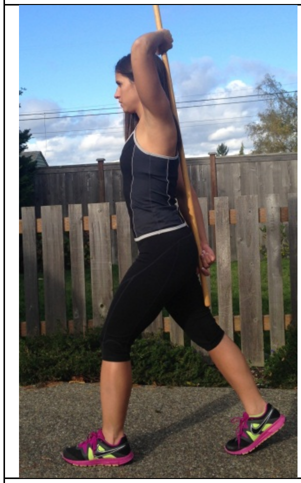</td><td>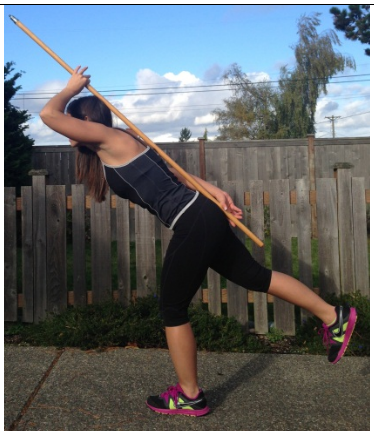</td></tr>
<tr><td colspan="2">1. Stand with affected leg forward and dowel along spine. Place head, shoulders and hips so that they are flush along the dowel. You should be on the toes of your rear foot.
2. Lean forward, keeping the heads, shoulders and hips along the dowel .
3. Allow your rear toes to leave the ground approximately 12 inches, still maintaining proper alignment with the dowel.
Dosing Guidance: 3 SETS of 10 repetitions performed 3-4 TIMES/WEEK</td></tr>
</table>

SUPPLEMENTAL FILE B – Motor Control Exercises

Exercise Name: Lateral Step-down with Heel Hover (with resistive band)

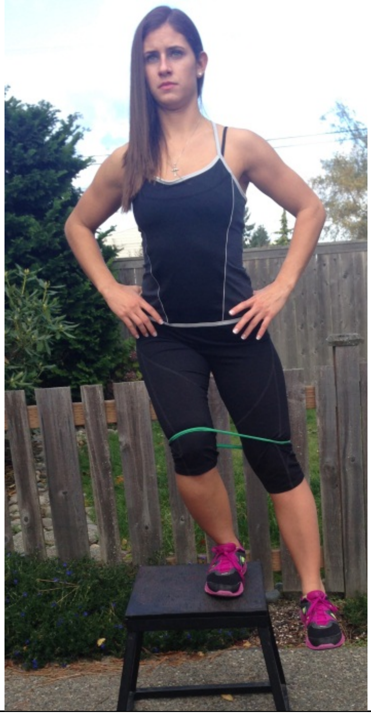

1. Stand with one foot on the edge of the box and the other leg unsupported. Place a black theraband around your thighs above your knees.
2. SLOWLY lower the unsupported leg, controlling your descent. Keep foot hovering; DO NOT TOUCH THE GROUND. Focus on keeping the supported knee in line with the second toe.
3. Do not allow the inside of your standing foot to raise off the box.

***Dosing Guidance*: 3 SETS of 10 repetitions performed 3-4 TIMES/WEEK**

Exercise Name: Side Plank

Modified: bottom knee bent	Standard: knees straight	Progression: top leg lift
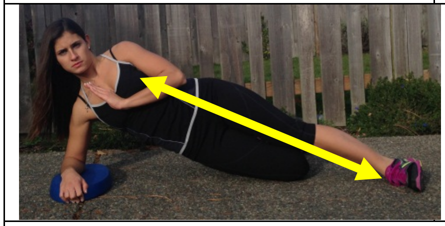	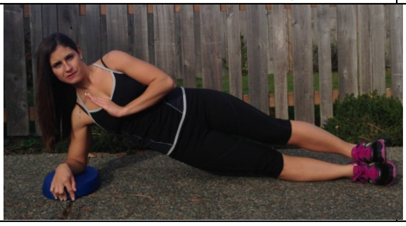	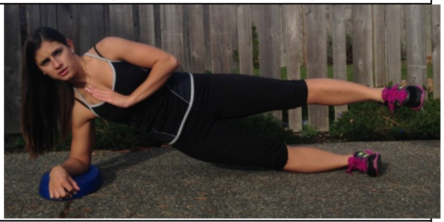

1. Modified: Lie on side, with the bottom knee bent. Prop weight through bottom elbow and extend top arm along side. Place thumb of the opposite hand on your sternum.
2. Slowly lift hips off of mat, keeping the core muscle activated. Keep head, shoulder, hip, knee and ankle all in one straight line, as shown above with the arrow.
3. Slowly control lowering hips back towards the mat.
4. Standard: Keep both knees straight.
5. Progression: Lift top leg after you lift your hips.

***Dosing Guidance*: 3 SETS of 10 repetitions performed 3-4 TIMES/WEEK**

SUPPLEMENTAL FILE B – Motor Control Exercises

Exercise Name: Seated Isometric Hip Flexion

Modified: Hand support under thigh	Progression: Without hand support
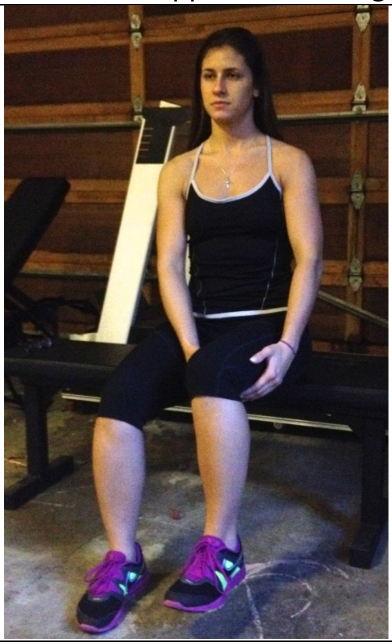	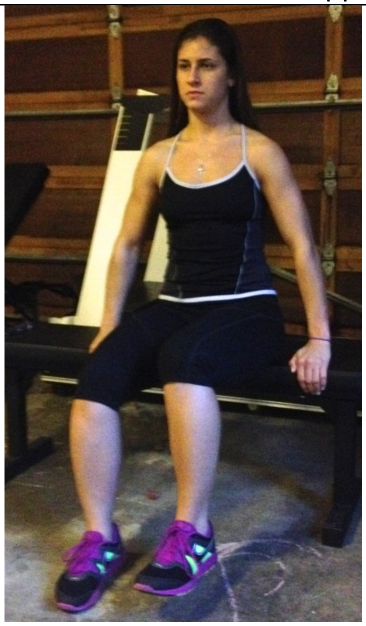

1. Sit on a bench or chair so that your hip is placed at 90 degrees of hip flexion (hip even with knee).
2. Modified: Place hands under thigh to help support your thigh as you raise your hip from 90 to 100 degrees of hip flexion (very small movement). Hold 1 second and progress to 5 seconds.
3. Progression: (after able to hold 5 sec at each position)
    a. Support up/hold/down
    b. Support up/down
    c. Support up
    d. No assist

***Dosing Guidance*: 3 SETS of 10 repetitions performed 3-4 TIMES/WEEK**

Exercise Name: Supine Hip Flexion with Theraband

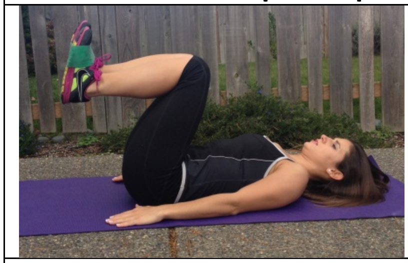	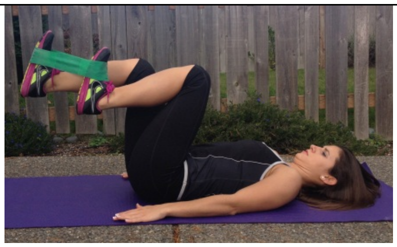	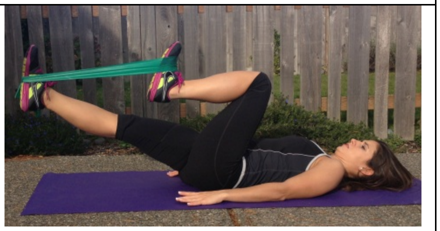
Start Position	End Position	Progression

1. Lie on your back with your knees and hips bent to 90 degrees. Place a tied theraband around the balls of your feet.
2. Simultaneously, pull your affected knee towards your chest approximately 10 degrees, while pushing the opposite leg away from you 10 to 12 inches (PAIN-FREE range). Avoid arching your lower back by tightening abdominals before each rep.
3. Progression: Push opposite leg further away, maintaining a neutral lower back.

***Dosing Guidance*: 3 SETS of 10 repetitions performed 3-4 TIMES/WEEK**
