# Supplementary material for: Two-year outcomes after arthroscopic surgery compared to physical therapy for femoracetabular impingement: A protocol for a randomized clinical trial
Source: BMC Musculoskelet Disord. 2016 Feb 4;17:60. doi: 10.1186/s12891-016-0914-1 (PMC4743428; doi:10.1186/s12891-016-0914-1)
Supplement: Additional file 5: — Physical Therapy Program—Mobility and Stretching Exercises. (PDF 609 kb) [file 12891_2016_914_MOESM5_ESM.pdf]

SUPPLEMENTAL FILE C: Mobility Exercises

Note: All subjects have provided their consent to appear in the figures.

Exercise Name: Kneeling Internal Rotation Self-Mobilizations with Lateral Distraction	
Starting Position	Ending Position
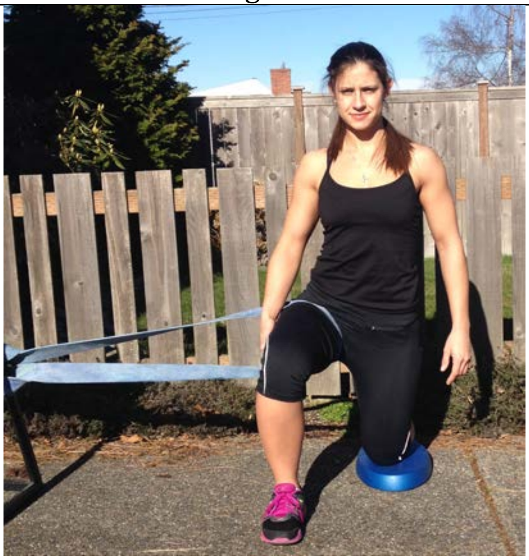	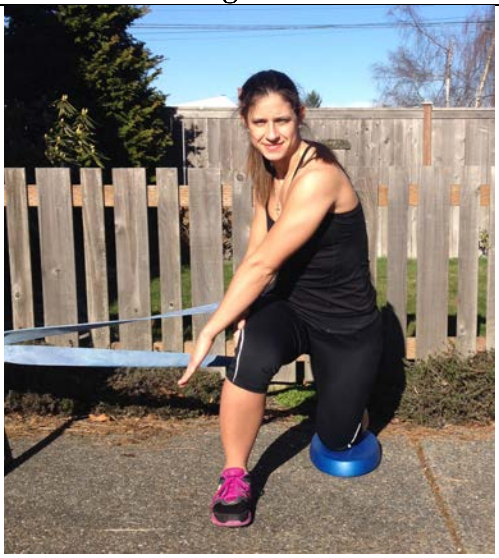

1. Position yourself in a half kneel position with your non-affected hip in the rear position.
2. Place your hand on the outside thigh of your front thigh near your knee for stabilization.
3. Keeping your gaze forward, reach forward with your back arm imagining that you are rotating the bony part of the pelvis of the rear leg moving toward the knee of the front leg.
4. Hold 1-2 seconds

Dosing Guidance: **2-3 SETS of 15 repetitions performed 1-2 TIMES/DAY**

Exercise Name: Half-Kneel FABER Self-mobilizations with Band	
Starting Position	Ending Position
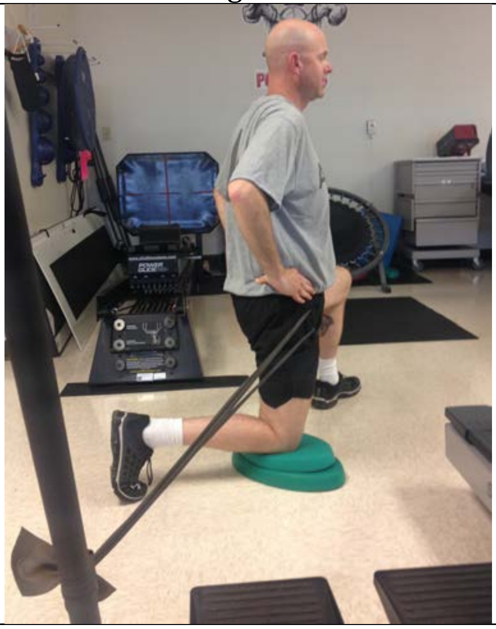	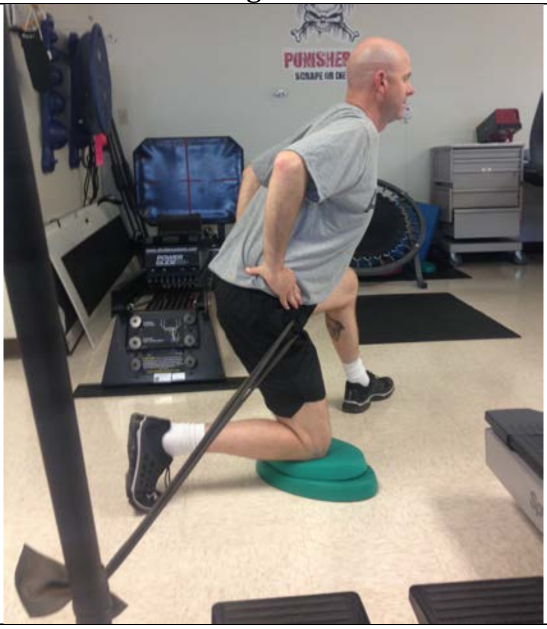

1. Position yourself in a half kneel position with your unffected hip kneeling in the rear position.
2. Place an anchored band on the outside of the affected thigh at a 45 degree angle behind you (this leg should be further away from the anchored position than the non-affected leg). Place your hands on your hips for stabilization.
3. Keeping your chest "puffed", hinge at the hips and then return to the start position. You should feel a stretch WITHOUT pain.

Dosing Guidance: **2-3 SETS of 15 repetitions performed 1-2 TIMES/DAY**

SUPPLEMENTAL FILE C: Mobility Exercises

**Exercise Name: Quadraped Rock Self-Mobilization with Lateral Distraction**	
Starting Position	Ending Position
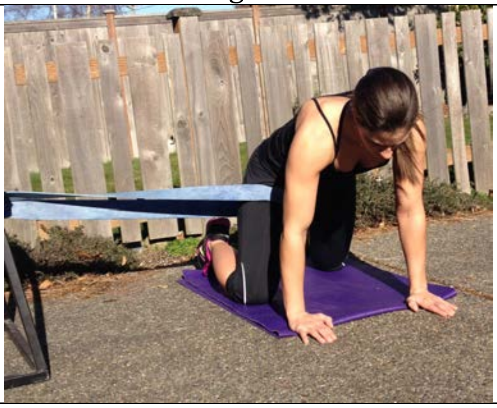	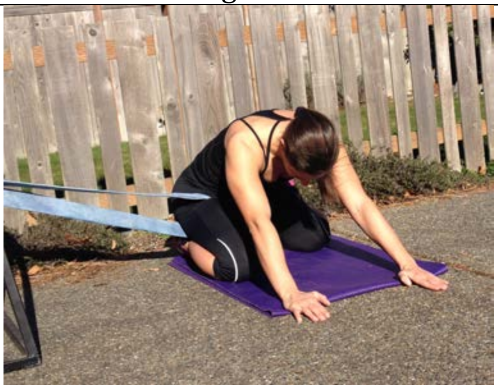

1. Position yourself on your hands and knees with your painful hip staggered forward 6-8 inches from your non-painful hip.
2. Anchor the band and place on the inside of your painful hip as close to your groin as possible. You may need a towel between the band and your thigh for comfort.
3. Shift your weight towards your unaffected side and then rock your hips back towards your heels in your PAIN-FREE range.
4. Hold 1-2 seconds.

Dosing Guidance: **2-3 SETS of 15 repetitions performed 1-2 TIMES/DAY**

Exercise Name: Prone Figure-4 Self-Mobilization

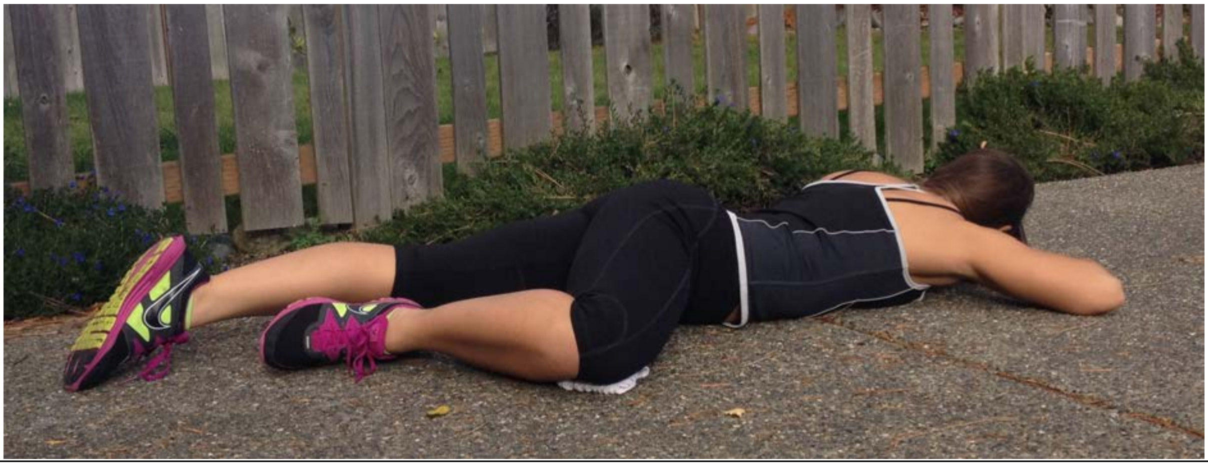

1. Lie in a prone figure-4 position as demonstrated in the above picture.
2. Place rolled up towel under thigh just above the knee.
3. Move the bony part of the front of your hip towards the table gently until you feel a stretch in the front of your hip. This is a VERY small movement. Avoid allowing your lumbar spine to move.
4. Hold 1-2 seconds.

Dosing Guidance: **1 SET of 10 repetitions performed 2-3 TIMES/DAY**

SUPPLEMENTAL FILE C: Mobility Exercises

Exercise Name: ITB Soft Tissue Self-Mobilization on Foam Roll

Bend Knee	Straighten knee
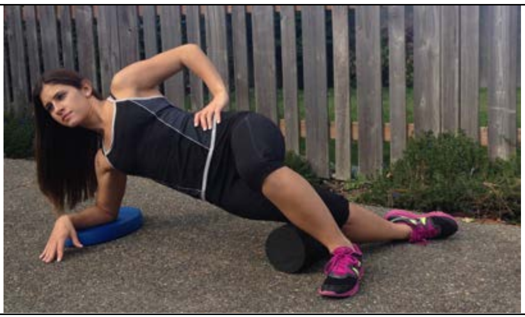	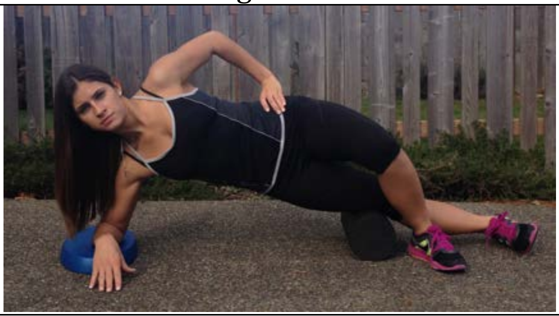

- Lie on side so that hip is directly placed onto foam roll. Keep the bottom leg straight and bend top knee so that the bottom of your foot is on the ground.
- Place forearm on the floor for upper body support and other hand on hip.Keep body straight as you move your body along the foam roll so the roll moves from hip to knee.
- Pause and breathe in then out at each place of discomfort as you roll. After you take a breath, bent and straighten your knee 1-3 times. Perform for 60 seconds.

Dosing Guidance: **1 SET of 3 repetitions performed 1-2 TIMES/DAY**

Exercise Name: Quadriceps Soft Tissue Self-Mobilization on Foam Roll

Bend Knee	Straighten knee
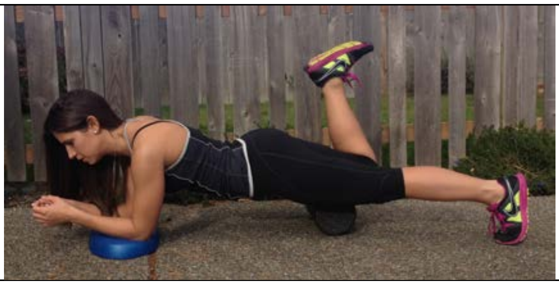	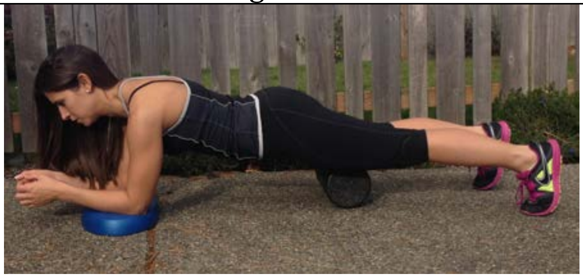

- Position yourself in a plank so that the front of your thigh is placed onto foam roll with one thigh on the foam roll and the other thigh off the edge of the foam roll.
- Pause and breathe in then out at each place of discomfort as you roll. After you take a breath, bent and straighten your knee 1-3 times. Perform for 60 seconds.

***Dosing Guidance*:** **1 SET of 3 repetitions performed 1-2 TIMES/DAY**

Exercise Name: Piriformis/Glut Min Self Myofascial Release on Ball

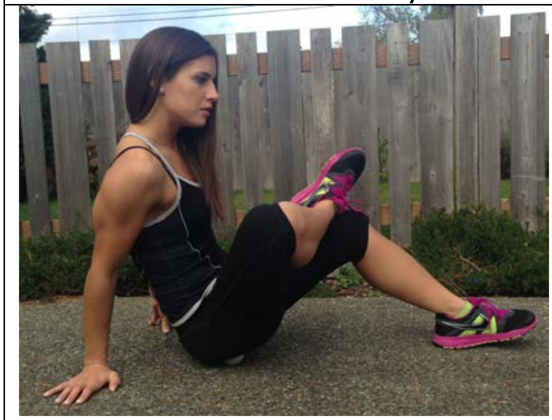

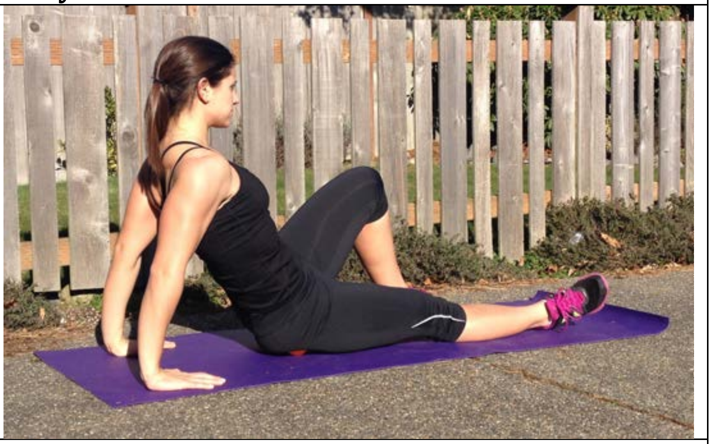

- Piriformis: Sit on lacrosse ball and cross the affected leg in figure-4 position over opposite leg.
- Glut min**:** Sit on lacrosse ball with a straight knee and toes turned slightly outwards. The opposite knee should be bent to 90 degrees.
- Place your hands behind you for upper body support. Locate the painful spot and either remain stationary or perform small rolls over painful area. Perform for 60 seconds.

Dosing Guidance: **1 SET of 3 repetitions performed 1-2 TIMES/DAY**

Exercise Name: Standing Figure-4 Stretch

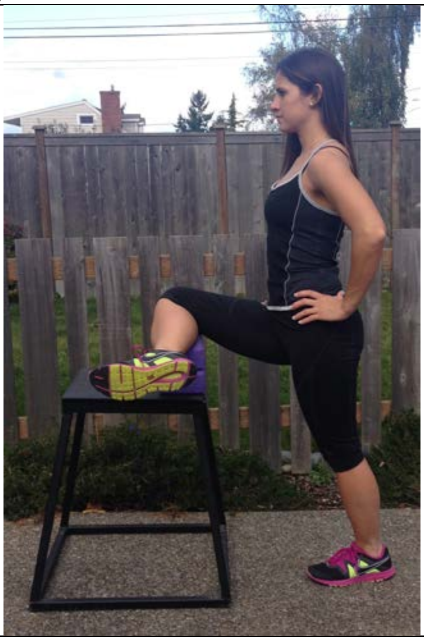

- Place affected side in figure 4 position onto high table or stool. Place support under knee and lower leg in order to have lower leg parallel to the ground.
- Completely straighten the rear leg and point toe directly forward.
- Pull in abdominals so that spine is completely stretch (AVOID arched back).
- Progression: Place rear leg further behind you.
- Hold 30 seconds.

***Dosing Guidance:* 1 SET of 2 repetitions performed 1-2 TIMES/DAY**

Exercise Name: Kneeling Tri-Planar Mobilizations

Forward	Backward	Clockwise	Counter-Clockwise
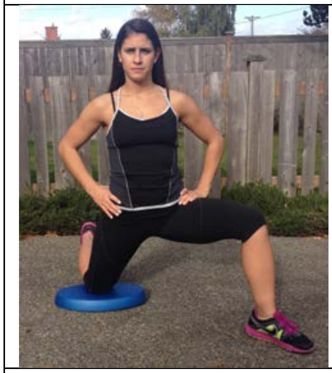	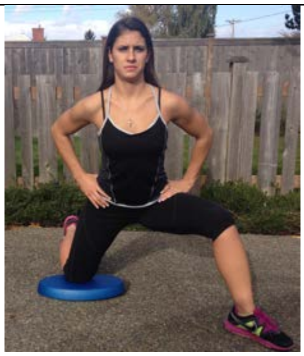	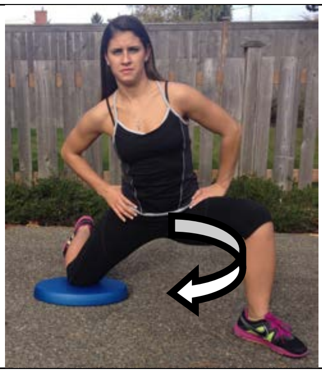	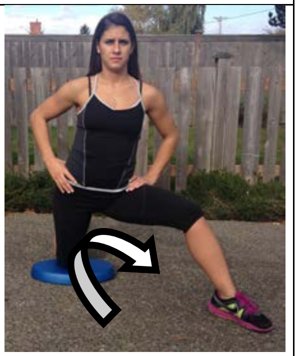

- Start with affected leg bent to 90 deg hip flex and rotated outwardly in PAIN-FREE range.
- Move knee forward towards toe to end range and then back off halfway. This is your starting position.
- Move hips back and forth 10 times in this position in PAIN-FREE range.
- Reset your starting position and then perform clockwise hip circles in PAIN-FREE range.
- Reset your starting position and then perform counter- clockwise hip circles in PAIN-FREE range.
- Switch and perform the above on the other side.

***Dosing Guidance*: 1 SET of 10 repetitions in each direction, performed 1-2 TIMES/DAY**
